# Brain‐wide mapping of inputs to the mouse lateral posterior (LP/Pulvinar) thalamus–anterior cingulate cortex network

**DOI:** 10.1002/cne.25317

**Published:** 2022-04-06

**Authors:** Yi Ning Leow, Blake Zhou, Heather A. Sullivan, Alexandria R. Barlowe, Ian R. Wickersham, Mriganka Sur

**Affiliations:** ^1^ Department of Brain and Cognitive Sciences Picower Institute for Learning and Memory Massachusetts Institute of Technology Cambridge Massachusetts USA; ^2^ McGovern Institute for Brain Research Massachusetts Institute of Technology Cambridge Massachusetts USA

**Keywords:** brain mapping, frontal cortex, lateral thalamic nuclei, neural pathways, pulvinar, superior colliculi

## Abstract

The rodent homolog of the primate pulvinar, the lateral posterior (LP) thalamus, is extensively interconnected with multiple cortical areas. While these cortical interactions can span the entire LP, subdivisions of the LP are characterized by differential connections with specific cortical regions. In particular, the medial LP has reciprocal connections with frontoparietal cortical areas, including the anterior cingulate cortex (ACC). The ACC plays an integral role in top‐down sensory processing and attentional regulation, likely exerting some of these functions via the LP. However, little is known about how ACC and LP interact, and about the information potentially integrated in this reciprocal network. Here, we address this gap by employing a projection‐specific monosynaptic rabies tracing strategy to delineate brain‐wide inputs to bottom‐up LP→ACC and top‐down ACC→LP neurons. We find that LP→ACC neurons receive inputs from widespread cortical regions, including primary and higher order sensory and motor cortical areas. LP→ACC neurons also receive extensive subcortical inputs, particularly from the intermediate and deep layers of the superior colliculus (SC). Sensory inputs to ACC→LP neurons largely arise from visual cortical areas. In addition, ACC→LP neurons integrate cross‐hemispheric prefrontal cortex inputs as well as inputs from higher order medial cortex. Our brain‐wide anatomical mapping of inputs to the reciprocal LP‐ACC pathways provides a roadmap for understanding how LP and ACC communicate different sources of information to mediate attentional control and visuomotor functions.

## Graphical abstract

We perform projection‐specific monosynaptic mapping of inputs to the reciprocal LP and ACC network in mice and show that the LP‐ACC network integrates information from regions which parallel that of the primate pulvinar‐prefrontal network. LP→ACC and ACC→LP neurons share common sources of inputs from regions involved in orienting and sensorimotor control. Inputs to the ACC→LP projection may serve to provide spatial context or visuomotor instructions, while the bottom‐up LP→ACC circuit integrates multisensory information and could serve as a route for SC feedback to frontal cortex. Our study serves as a primer for future circuit‐level examination of LP‐ACC interactions and inputs integrated by this circuit.

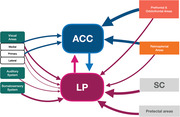

## 1 INTRODUCTION

The rodent homolog of the primate pulvinar, the lateral posterior (LP) thalamic nucleus, is a higher order visual thalamic structure that shares reciprocal connectivity with multiple visual, associational, and frontal cortical areas (Adams et al., 2000; Juavinett et al., 2019; Scholl et al., 2020). The pulvinar/LP has been implicated in a broad range of functions that extend beyond visual processing, including conveying saccade‐related activity, coordinating visually guided movements, and mediating spatial attention (Kaas & Lyon, 2007; Kastner et al., 2020; Robinson et al., 1986). Like the primate pulvinar, rodent LP is organized into different subregions, each with preferential inputs from distinct subcortical and cortical areas along the visual hierarchy (Bennett et al., 2019). LP has at least two subdivisions, each containing a retinotopic map of visual space, with the posterior LP (pLP) driven by superficial layer superior colliculus (SC) visual inputs and the anterior LP (aLP) driven by visual inputs from the primary visual cortex (VISp) (Beltramo & Scanziani, 2019; Bennett et al., 2019). A third LP subregion, medial LP (mLP), has also been defined by its reciprocal connectivity with frontal areas, including the anterior cingulate cortex (ACC) and the orbito‐frontal cortex (OFC) (Bennett et al., 2019). However, little is known about the inputs to mLP‐frontal cortex neurons and how these might mediate the functions of this circuit.

The medial pulvinar is one of the thalamic structures that has undergone the greatest expansion during evolution (Baldwin et al., 2017; Kaas & Baldwin, 2019; Rosenberg et al., 2008). Medial pulvinar connections with frontal and premotor areas are likely involved in higher order visual cognition rather than early visual processing (Homman‐Ludiye & Bourne, 2019; Homman‐Ludiye & Bourne, 2021; Romanski et al., 1997). Mutual interactions between the pulvinar and the frontal‐parietal attention network regulate sustained and selective attentional allocation during visual decision making (Rafal & Posner, 1987; Snow et al., 2020; Kastner et al., 2020). Lesions or inactivation of the primate dorsal/medial pulvinar appear to impair visual decision making, but performance in visual tasks remains intact following manipulations to the tasks' incentive structure and presumably attentional allocation (Komura et al., 2013; Wilke et al., 2013), suggesting that inactivating medial pulvinar does not impair simple visual processing. Furthermore, deficits in grasping and proprioception following dorsal‐medial pulvinar lesions have also been attributed to pulvinar interactions with premotor areas and primate frontal eye fields (FEFs) (Trojanowski & Jacobson, 1974; Wilke et al., 2018), which are likely responsible for coordinating motor strategies for orienting toward attended targets.

Attentional regulation and sensorimotor integration are complex cognitive operations involving the integration of information from more than just the visual areas. Yet, our understanding of the inputs integrated by the pulvinar remains mostly restricted to its interaction with visual structures and has been largely cortico‐centric. Consequently, understanding of the brain‐wide and multimodal influences on pulvinar/LP circuits is limited, particularly in rodents. Although the medial pulvinar has been thought to be exclusive to primates (Homman‐Ludiye & Bourne, 2019; Rosenberg et al., 2008), the identification of a homologous rodent medial LP subregion with frontal cortex interconnectivity (Bennett et al., 2019) suggests that rodent medial LP could have comparable brain‐wide interactions as primates to subserve potentially similar roles in sensorimotor integration and spatial attention. Furthermore, while the fronto‐parietal networks typical of primates are less prominent in rodents, the rodent ACC and secondary motor areas (MOs) have been associated with comparable sensorimotor functions. Notably, a region of the rodent ACC/MOs has been described as rodent “frontal orienting field” (FOF), in putative homology to the primate FEF, given the ACC/MOs area's extensive connectivity with the SC and the visual areas (Zingg et al., 2014, but see Preuss & Wise, 2021), as well as its functional involvement in controlling eye movements (Sato et al., 2019) and other orienting actions (Ebbesen et al., 2018; Huda et al., 2020).

Inputs from the rodent ACC/MOs region to the SC have also recently been shown to control orienting and motor biases (Huda et al., 2020), and modulate visual perception (Hu et al., 2019). Indeed, the fronto‐parietal areas, the SC, and the pulvinar seem to constitute an integrated network critical for attentional selection (Baleydier & Mauguiere, 1985; Snow et al., 2009). However, in spite of the top‐down influence of ACC/MOs upon the SC, the SC does appear not send any direct input back to ACC/MOs. As with the pulvinar, the rodent LP can potentially serve as one of the relays for SC input back to the frontal areas. It remains an open question whether rodent LP connectivity with the ACC/MOs region could parallel the SC‐pulvinar‐frontal/FEF networks described in primates (Baleydier & Mauguiere, 1985; Homman‐Ludiye & Bourne, 2019; Homman‐Ludiye et al., 2019; Romanski et al., 1997; Wilke et al., 2018). To address this gap, we sought to delineate the anatomical network in which the rodent medial LP is embedded, focusing on its connections with the ACC. Specifically, we comprehensively mapped brain‐wide inputs to reciprocal nodes of the LP→ACC circuit, in order to understand: (1) the nature of inputs integrated by LP, which influences its eventual output to higher order areas, such as the ACC, and (2) inputs influencing top‐down feedback from ACC to LP, which can serve as a conduit for modulating visual or sensorimotor processing (Hu et al., 2019). We show that the sources of inputs integrated by medial LP and ACC are comparable to those of the medial pulvinar described in primates (Baleydier & Mauguiere, 1985; Romanski et al., 1997; Rosenberg et al., 2008). From the different complement of sources, we suggest that the top‐down ACC→LP projection may serve to provide spatial context or visuomotor instructions, while the bottom‐up LP→ACC circuit integrates multisensory information and could serve as a route for SC feedback to frontal cortex.

## 2 MATERIALS AND METHODS

### 2.1 Mice and surgery procedures

All experiments were performed under protocols approved by the MIT Institutional Animal Care and Use Committee and conformed to NIH guidelines. A total of 20 C57BL/6J wild‐type mice and four Vgat1‐Cre mice were used in these experiments, with six mice excluded due to lack of fluorescent labeling in starter cell region. Mice of both sexes were used in this study. All mice were group‐housed on a 12‐h reversed light‐dark cycle before and after surgeries.

### 2.2 Viral injections and rabies tracing

Throughout stereotaxic surgeries, mice were maintained anesthetized using isofluorane (4% for induction and 1% for maintenance) while head‐fixed on a stereotaxic frame. Viral injections were performed using glass micropipettes and injected at a rate of 50–75 nl/min. After injection, micropipettes were slightly withdrawn by 0.05 mm and then left in place for 10–15 min after injection to minimize nonspecific diffusion of the virus along the needle track. After each surgery, the scalp was tightly sutured and mice recovered on heated water‐circulation pad.

To map reciprocal inputs between LP and ACC, anterograde and retrograde tracers were simultaneously injected into medial LP in a 1:1 mixture (AP: −1.9, ML: 1.2, DV: −2.65 mm from Bregma) of retroAAV‐hSyn‐mCherry and AAV1‐hSyn‐eYFP (150 nl total). To confirm that medial LP neurons projected to ACC, CTB‐Alexa Fluor 488 was injected in ACC (AP: +0.75, ML: 0.3, DV: 100 nl each at 1 and 0.5 mm from pial surface).

To trace inputs to projection‐specific populations, we used a monosynaptic rabies tracing strategy where only cells projecting to a target population would express proteins necessary for rabies virus infection and retrograde transsynaptic spread (Lavin et al., 2019; Wickersham et al., 2006). Briefly, Cre‐recombinase‐dependent transcription of helper proteins for rabies infection and transynaptic spread allow projection‐specific targeting when Cre recombinase is present in the starter cell region, which can be delivered by a retrograde AAV in the projection target. For LP→ACC projections, retroAAV‐Cre (Addgene: 105553‐AAVrg, 2.1 × 10^13^ genome copies [gc]/ml) was first injected in ACC (AP: +0.8, ML: 0.3 mm from Bregma, 150 nl each at DV: 1.25 and 0.75 mm from pial surface). AAV helper viruses contained a 1:1 mixture of AAV‐syn‐FLEX‐splitTVA‐EGFP‐tTA (Addgene: 100798‐AAV1, 7.6 × 10^10^ gc/ml) and AAV1‐TREtight‐mTagBFP2‐B19G (Addgene: 100799‐AAV1, 6.5 × 10^11^ gc/ml), each diluted to viral titers as previously described (Lavin et al., 2019), and were injected in medial LP (150 nl; AP: −1.8, ML: 1.2, DV: 2.6 mm from Bregma). After a week to allow for viral expression, G‐deleted rabies virus (200 nl; RVΔG‐4mCherry (EnvA), 9.53 × 10^10^ infectious units/ml) was injected into the same LP site. For inputs to ACC→LP projections, retroAAV‐Cre was injected in medial LP. AAV helper viruses were injected in ACC (AP: +0.8, ML: 0.5 mm from Bregma, 300 nl at DV: 1 mm from pial surface), followed by rabies virus (300 nl) at the same site a week after AAV injections.

In control experiments to test for cre‐independent expression of viral transgenes, we performed all above except injecting the same volume of sterile 0.9% NaCl instead of retroAAV‐cre. To test for rabies infection‐independent labeling, an AAV construct without the G protein, AAV1‐TREtight‐mTagBFP2 (∼6.5 × 10^11^ gc/ml), was used instead of AAV1‐TREtight‐mTagBFP2‐B19G.

For mapping inhibitory inputs to LP, 100 nl of retroAAV‐hSyn‐mCherry (Addgene: 50459‐AAVrg, 1 × 10^13^ gc/ml) was injected into LP (AP: −1.9, ML: 1.2, DV: −2.6 mm from Bregma) of Vgat1‐Cre mice.

### 2.3 Histology and immunohistochemistry

Mice were deeply anesthetized before transcardial perfusion with 4% paraformaldehyde (PFA) a week after rabies virus injection. The brains were kept in 4% PFA overnight, and then transferred to phosphate buffered saline for storage at 4°C until sectioning. Hundred micrometers thick coronal sections were prepared on a vibratome (VT1200S, Leica). With the exception of 8–10 slices in sections containing starter cells (for LP→ACC inputs—AP relative to Bregma approximately: −1.6 to −2.3 mm; for ACC→LP inputs—AP: +0.5 to +1.2 mm), sections were mounted upon slicing with Vectashield mounting medium with DAPI. Starter cell slices from each brain sample were kept for further immunohistochemistry to amplify the green fluorescent protein (GFP) signal in regions containing starter cells. Sections were stained with the primary antibody (Chicken anti‐GFP) at 1:500 concentration (overnight at 4°C), and subsequently the secondary antibody (Donkey anti‐chicken Alexa Fluor 488‐conjugate) at 1:200 concentration (4 h at room temperature).

### 2.4 Confocal imaging

Slices were imaged with a Leica TCS SP8 confocal microscope using a 10X / 0.40 NA objective lens at 2X zoom. Full slices were acquired by tiling reconstruction through the Leica Application Suite.

### 2.5 Image and data analysis

Slices were manually aligned to templates from the Allen Reference Atlas (Mouse, Adult, 3D Coronal). Cells positive for mCherry outside of the starter cell region were all manually counted and assigned to regions as defined in the Allen Reference Atlas following alignment. A small number of labeled cells (< 10 per brain) with astrocytic morphology were excluded from input cell counts. For each brain sample, the number of cells in a region was normalized as a percentage of all counted cells outside the starter cell region. We were unable to identify GFP‐containing starter cells due to the low viral titer optimized for dual AAV transfection. Thus, after verifying that injections were targeted to desired nuclei (LP and ACC) indicated by injection needle track, we define the starter cell region as the full starter region (whole LP for LP→ACC input tracing or whole ACC for ACC→LP input tracing) as determined after atlas alignment. Plots were generated with matplotlib (Python) and figures assembled on Adobe Illustrator. After manual cell counts, percentage of input from each region was determined as a proportion of all input cells within the single brain sample. The proportion of input from each brain sample was then averaged across subjects and plotted as a group average.

## 3 RESULTS

### 3.1 Anatomical gradients of reciprocal LP→ACC circuits

**FIGURE 1:**
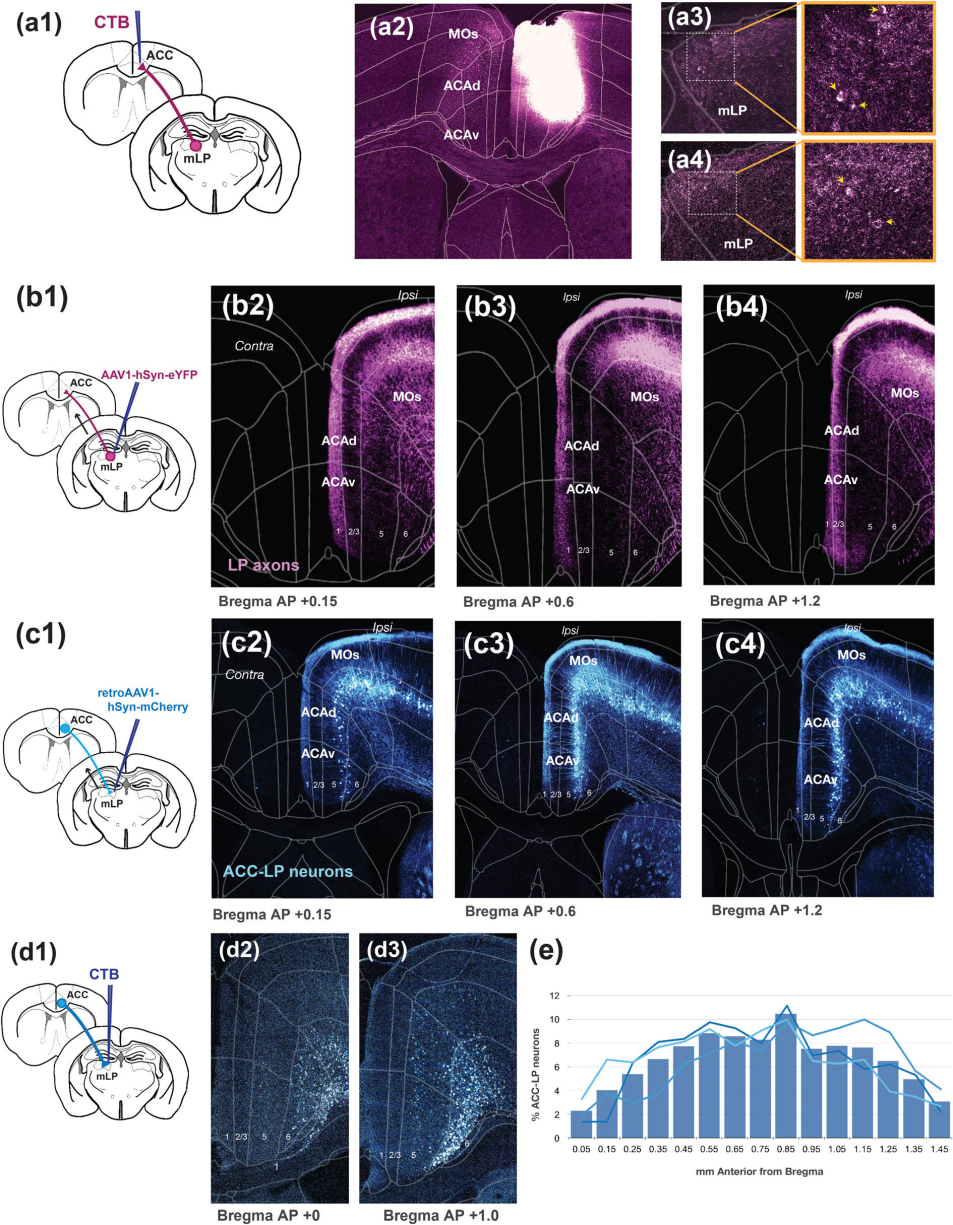
Anatomical gradients of reciprocal LP→ACC circuits. (a1) Retrograde labeling from ACC with fluorophore‐conjugated CTB. (a2) Injection site in ACC. (a3 and a4) CTB retrogradely labeled neurons in LP found in medial subregions. (b1) Anterograde labeling of LP axons in ACC, with injection in medial LP. (b2–b4) LP axons in ACC spanning (b2) +0.15, (b3) +0.6, and (b4) +1.2 mm anterior from Bregma. (c1) Retrograde labeling of LP‐projecting ACC neurons, with retrograde AAV injection in medial LP. (c2–c4) ACC neurons projecting to LP spanning (c2) +0.15, (c3) +0.6, and (c4) +1.2 mm anterior from Bregma. (d1) Retrograde labeling of LP‐projecting ACC neurons, with CTB injection in medial LP. ACC neurons projecting to LP at (d2) +0 and (d3) +1.0 mm anterior from Bregma. (e) Quantification of ACC→LP projecting neurons along the rostrocaudal axis shows peak projection density at about 0.75–0.85 mm anterior to Bregma. Bars represent mean of *n* = 3 mice. Individual lines represent samples from each mouse

First, to examine the location of ACC‐projecting LP neurons, we injected the retrograde tracer CTB in both rostral and caudal ACC (see below). Retrogradely labeled LP→ACC neurons were densest in medial LP although sparser labeling of LP→ACC neurons was also observed in lateral areas of LP (Figure 1a). Consistent with previous descriptions of LP connectivity (Bennett et al., 2019), we found that LP→ACC neurons were mostly located in anterior sections of medial LP. With the location of LP→ACC neurons being densest about −1.8 mm posterior of Bregma, we used this to guide subsequent LP injections. Injecting an anterograde tracer (AAV1‐hSyn‐eYFP) in medial LP, we found LP axons throughout the rostro‐caudal axis of the ACC and secondary motor area (MOs) (Figure 1b). Thus, a relatively discrete region of LP provides the majority of inputs that span a large area of the ACC/MOs cortex.

Retrograde tracing from LP with retroAAV‐mCherry (Figure 1c) and CTB (Figure 1d) shows that ACC neurons project to LP from both layers 5 and 6. The rodent ACC spans a broad extent along the rostral‐caudal axis, which may contain anatomical and functional “gradients” (van Heukelum et al., 2020) or even distinct areas. For example, caudal ACC is more likely than rostral ACC to receive primary visual cortex (VISp/V1) input, while rostral rather than caudal ACC receives greater proportion of input from medial higher visual cortices (Huda et al., 2020). As these anatomical gradients can influence the information integrated by ACC neurons, we sought to identify where along the rostrocaudal axis the ACC neurons that project to LP predominate. We injected retrograde AAV carrying fluorescent mCherry in medial LP and quantified retrogradely labeled layer 5 and layer 6 ACC neurons along the anterior‐posterior axis (Figure 1c). We found LP‐projecting ACC neurons all along the rostral‐caudal axis, from 0 to +1.5 mm from Bregma, with peak density at about +0.8 mm (Figure 1d). Thus, the peak of ACC→LP neurons did not strictly correspond to rostral or caudal ACC—and may instead reflect the integrative nature of ACC input to LP. Notably, the peak density of ACC→LP projections also included areas of ACC/MOs that have been described as the rodent FOF (Ebbesen et al., 2018; Zingg et al., 2014).

### 3.2 Mapping of inputs integrated by ACC→LP neurons and LP→ACC neurons

**FIGURE 2:**
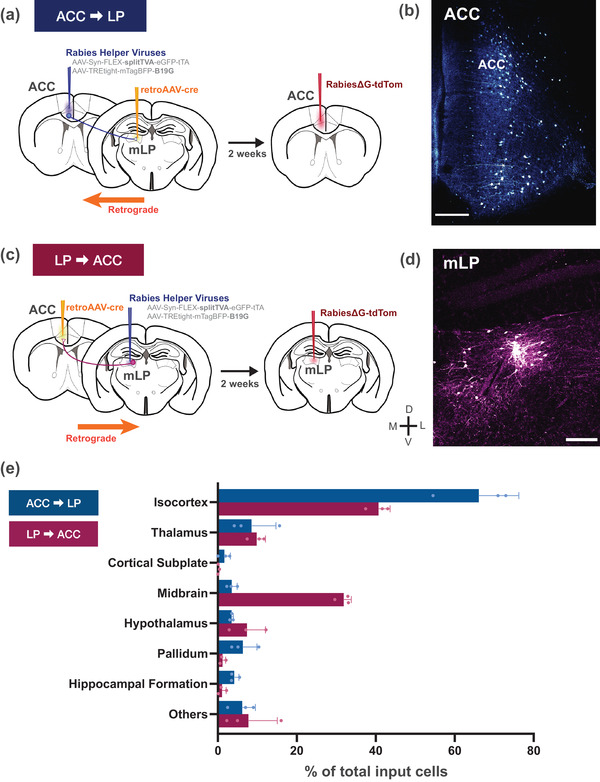
Whole‐brain input tracing to ACC→LP neurons and LP→ACC neurons. (a) Rabies viral tracing strategy for ACC→LP neurons involved retrograde transport of Cre recombinase injected in LP. Helper AAVs were injected in ACC for Cre‐dependent expression of rabies helper proteins. After a week, G‐deleted rabies virus was injected in ACC. (b) Starter cell region (ipsilateral ACC) for ACC→LP input mapping. (c) Rabies viral tracing strategy for LP→ACC neurons. Retrograde‐AAV carrying Cre recombinase was injected in ACC, while helper viruses were injected in LP. G‐deleted rabies virus was injected in LP a week after AAV injections. (d) Starter cell region (ipsilateral LP) for LP→ACC input mapping. (e) Overview of brain regions projecting to ACC→LP (blue) and LP→ACC (magenta) neurons. Bars represent mean of *n* = 3 mice, error bars show the standard deviation, and circles represent samples from individual mice

We sought to map monosynaptic inputs specifically to ACC neurons projecting to LP (Figure 2a,b) and LP neurons projecting to ACC (Figure 2c,d), using a projection‐specific monosynaptic rabies tracing approach (Wickersham et al., 2007; Lavin et al., 2019). The tracing strategy involved injecting retrograde AAV carrying Cre recombinase (retroAAV‐cre) in the postsynaptic target region, LP or ACC (Figure 2a,c). The retrograde transport of Cre‐recombinase to the source of the projection facilitated the Cre‐dependent transcription of transgenes encoding helper proteins necessary for rabies infection (TVA receptor) and transsynaptic spread (rabies G protein). G‐deleted rabies virus was then injected at the site of projection, entering starter cells expressing TVA receptors but only spreading transynaptically from starter cells containing G. After a week to allow rabies virus transsynaptic spread, we characterized exogenous inputs to each of the projector populations by quantifying all rabies‐infected cells (mCherry‐positive) outside of the starter cell region.

**FIGURE 3:**
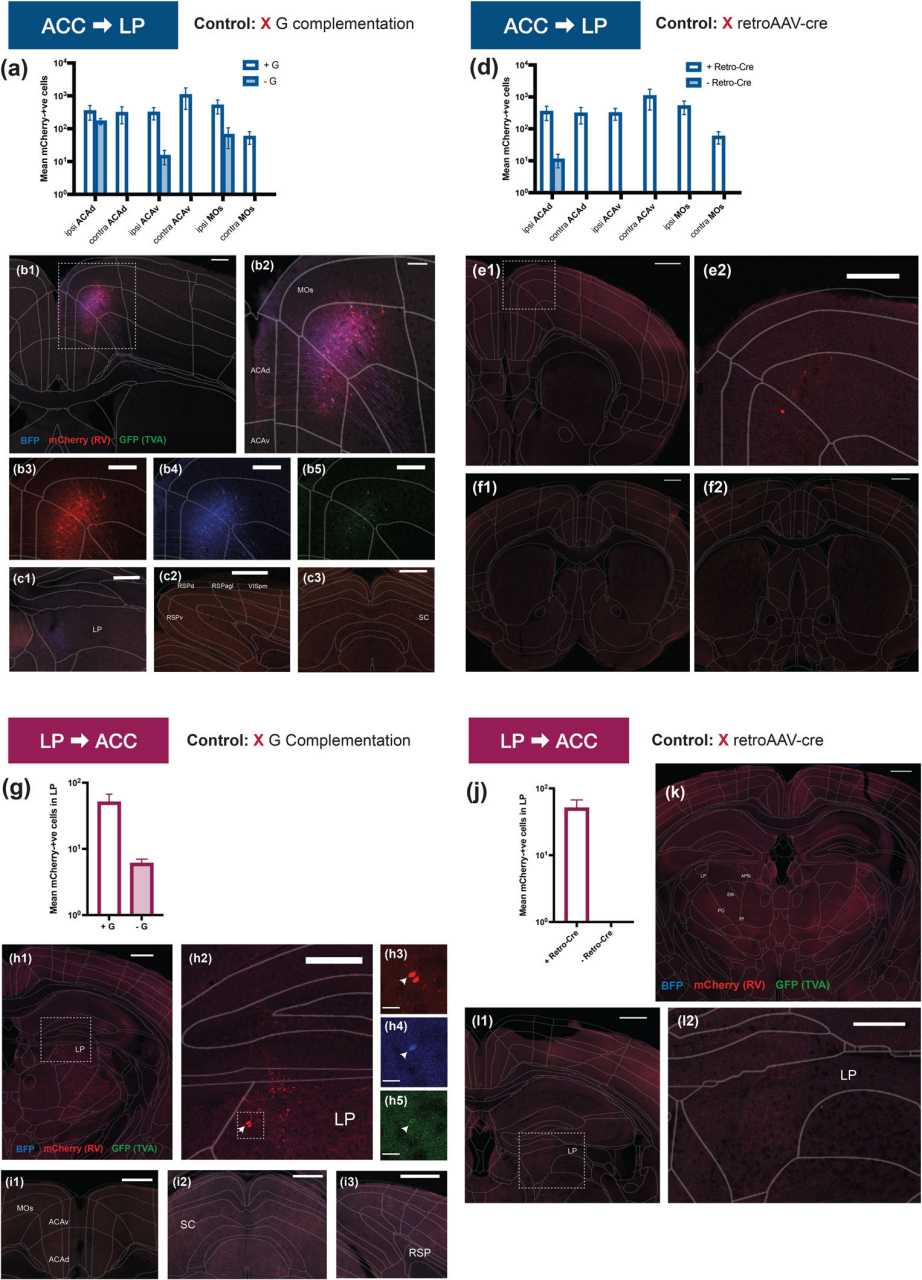
Control experiments lacking G complementation or lacking cre recombinase for rabies tracing of inputs. (a–c) No G‐complementation control for mapping inputs to ACC→LP neurons. (a) Quantification of rabies‐infected (mCherry‐positive cells) in ACA region in the experiment (+ G, *n* = 3) and control (– G, *n* = 2). Error bars indicate standard deviation. (b1) Labeled cells found in ACA and MOs but not in adjacent cortical regions. Scale bar = 250 μm. (b2) Magnified region in (b1). Scale bar = 125 μm. Magnified region of (b1) and (b2) with individual fluorescent channels: (b3) mCherry (rabies‐infected) (b4) BFP, and (b5) GFP (TVA). Without G protein complementation, there were no cells found in regions other than ACA and MOs, including (c1) LP, (c2) RSP, and (c3) SC. (d–f) No retroAAV‐Cre Control for mapping inputs to ACC→LP neurons. (d) Quantification of rabies‐infected (mCherry‐positive cells) in ACA region in the experiment (+ retro‐cre, *n* = 3) and control (– retro‐cre, *n* = 2). Error bars indicate standard deviation. Very few cells were found to have cre‐independent labeling with low if any leak expression of cre‐dependent transgenes. (e1) Micrographs showing a labeled cell found in ACAd. Scale bar = 500 μm. (e2) Magnified region in (e1). Scale bar = 250 μm. (f1 and f2) No labeled cells found in adjacent cortical regions and ACC regions. (g–i) No G‐complementation control for mapping inputs to LP→ACC neurons. (g) Quantification of rabies‐infected (mCherry‐positive cells) in LP in the experiment (+ G, *n* = 3) and control (– G, *n* = 2). Error bars indicate standard deviation. (h1) Labeled cells found in LP but not in any other regions. Scale bar = 500 μm. (h2) Magnified region in (h1). Scale bar = 250 μm. Magnified region of (h1) and (h2) with individual fluorescent channels: (h3) mCherry (rabies‐infected), (h4) BFP, and (h5) GFP (TVA). Without G protein complementation, there were no cells found in regions other than LP, including (i1) ACA and MOs, (i2) SC and (i3) RSP. (j–l) No retroAAV‐Cre Control for mapping inputs to LP→ACC neurons. (j) Quantification of rabies‐infected (mCherry‐positive cells) in LP in the experiment (+ retro‐cre, *n* = 3) and control (– retro‐cre, *n* = 2). Error bars indicate standard deviation. No cells were found to have cre‐independent labeling with low if any leak expression of cre‐dependent transgenes, (k) no rabies‐infected cells in all regions shown in section. Scale bar = 500 μm. (l1) No rabies‐infected cells found in mLP. Scale bar = 500 μm. (l2) Magnified region in (l1). Scale bar = 250 μm

**FIGURE 4:**
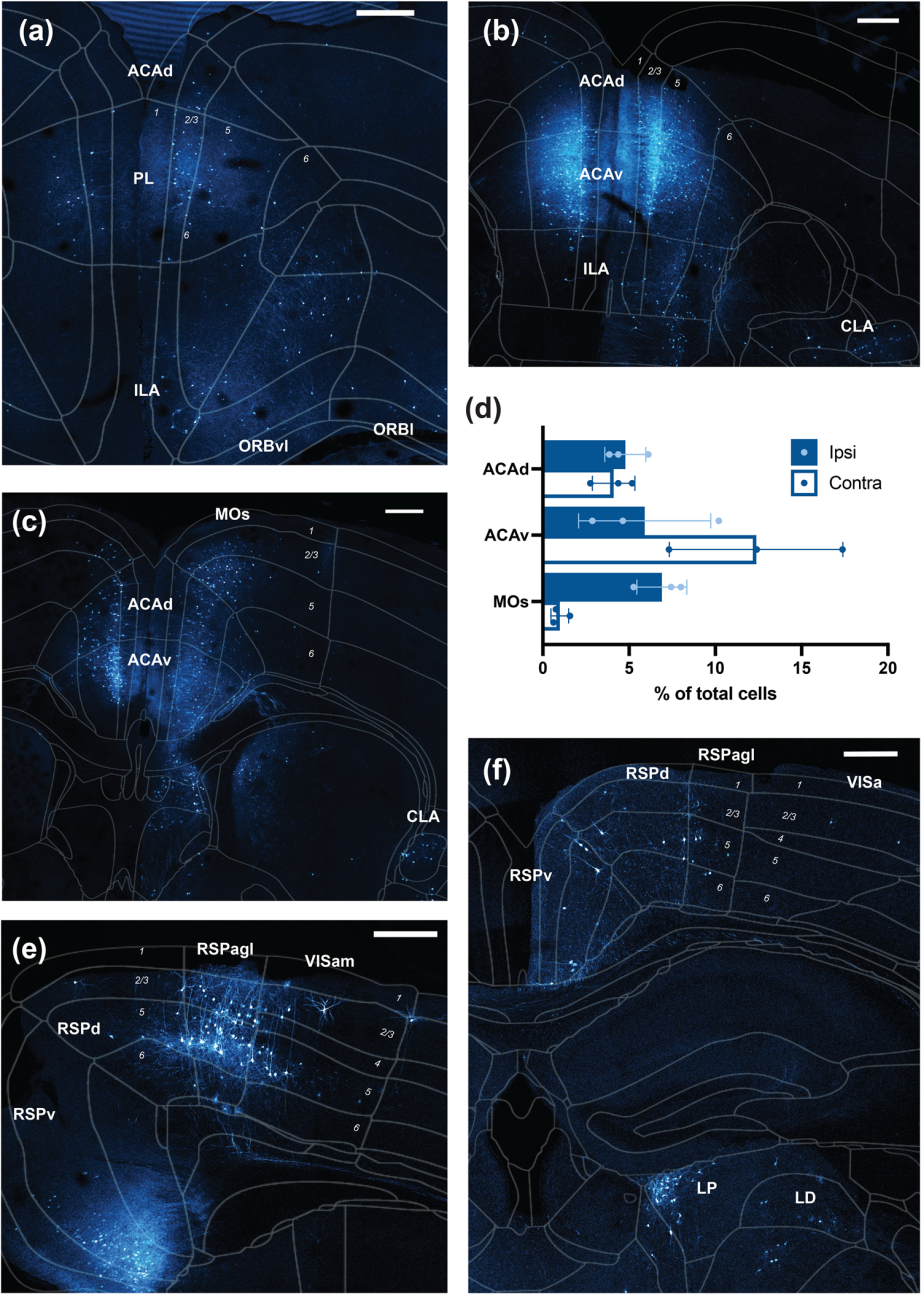
Frontal, prefrontal, and retrosplenial inputs to ACC→LP neurons. (a) Micrographs showing inputs from dorsal ACC, PL, ORB areas, and the agranular insula cortices. (b and c) Inputs to ACC→LP neurons include contralateral ACC, particularly ventral ACC. (d) Comparison of proportions of cells found in each hemisphere in ACA subregions and MOs, the values are taken as a percentage to all cells counted in each ACC→LP experimental animal. Bars represent mean of *n* = 3 mice, error bars indicate the standard deviation, and circles represent samples from individual mice. (E and f) Retrosplenial cortex inputs to ACC→LP neurons can arise from all retrosplenial subregions. Scale bars = 250 μm

Within starter cell regions, the LP contained an average of 45.3 mCherry‐labeled somas (putatively LP→ACC neurons). For ACC→LP input mapping, the ipsilateral ACC and MOs contained an average of ∼654 and ∼511 mCherry‐labeled somas, respectively. However, the number of ACC→LP neurons, or true starter cells, is expected to be much lower due to inclusion of cells in all layers and the strong recurrent local connectivity within ACC (Figure 4a–c). In control experiments where helper and rabies viruses were injected without retroAAV‐cre, an average of 11 rabies‐infected cells found in ipsilateral ACC were found in the ACC→LP control (Figure 3d–f), while 0 rabies‐infected cells were found throughout the brain in the LP→ACC control (Figure 3j–l), together indicating a very low rate of cre‐independent expression of helper proteins.

Due to difficulty in unambiguously distinguishing starter and input cells in ACC, we excluded all ACC and MO cells from the ipsilateral hemisphere from quantification of exogenous inputs. Quantifying brain‐wide exogenous inputs, we found an average of ∼7014 input cells to ACC→LP neurons (*n* = 3 mice) and ∼1657 input cells to LP→ACC neurons (*n* = 3 mice). In control experiments with no G protein complementation to facilitate transsynaptic spread of the rabies virus, we found rabies‐infected cells only limited to the starter cell regions—ipsilateral ACC/MOs for ACC→LP experiments (Figure 3b) and LP for LP→ACC experiments (Figure 3h). The absence of rabies‐infected cells in regions outside of the starter cell region in absence of G protein complementation suggests that input cells in the full experiments are likely true inputs to the LP→ACC and ACC→LP populations, and that artifactual labeling resulting from off‐target anterograde or retrograde transport of helper viruses is minimal if at all under our experimental conditions.

First, we sought to characterize the major brain areas sending input to these projection cells, delineating input regions as defined by the Allen Reference Atlas. An overview of the brain regions sending inputs to ACC→LP and LP→ACC neurons is shown in Figure 2e. Both ACC→LP and LP→ACC neurons received a substantial proportion of their input from the cortex. The isocortex made up approximately 62% of input to ACC→LP neurons, and 38% of input to LP→ACC neurons (Figure 2e). ACC→LP neurons received subcortical inputs from diverse sources throughout the brain, with the thalamus as the second dominant source of input to ACC→LP neurons (9.4% of input), followed by the pallidum (7.2%). In contrast, subcortical sources constituted a larger proportion of inputs to LP→ACC neurons (62%), approximately half of which originate from midbrain sources (29.9%).

### 3.3 Bilateral prefrontal and frontal cortex connectivity dominates inputs to ACC→LP neurons

**FIGURE 5:**
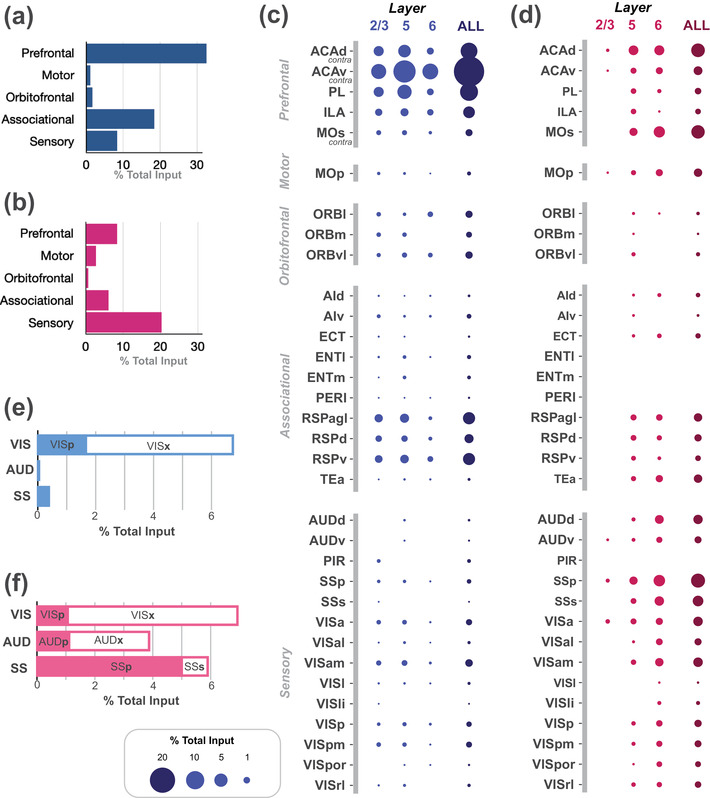
Cortical inputs to ACC→LP and LP→ACC projectors. (a and b) Distributions of the functional domains of cortical inputs to ACC→LP (A) and LP→ACC (B) neurons. (c and d) Cortical inputs to ACC→LP (c) and LP→ACC (d) neurons sorted by functional domains. These inputs are represented with a circle with radius proportional to the percentage of total inputs it represents for each layer, and also as a whole region with all layers summed (mean of *n* = 3 mice)

Consistent with the highly interconnected nature of prefrontal and frontal areas, we found that a large proportion of inputs (32.6% of total inputs and 52.5% of cortical inputs) to ACC→LP neurons were from the prelimbic (PL), infralimbic (ILA), and the orbitofrontal cortex (ORB) (Figures 4a–c and 5a). These areas sent input to ACC→LP neurons from both hemispheres, but predominantly from the ipsilateral cortex.

Outside of the ipsilateral ACC and MOs, rabies‐infected cells were found extensively in the contralateral hemisphere of these regions. The abundance of contralateral ACC neurons labeled reflects the strong recurrent connectivity within ACC and also the intra‐ACC interhemispheric information that ACC→LP neurons integrate. Interestingly, these contralateral ACC inputs to ACC→LP neurons were densest in the ventral subdivision of ACC (Figure 4c,d), which accounted for 12.6% of total inputs, and 56.8% of all contralateral cortical inputs to ACC→LP neurons. In contrast, contralateral ACAd and MO neurons represented 22.0% of total and 3.7% of all contralateral cortical inputs to ACC→LP neurons. In sum, these findings indicate that the top‐down ACC→LP projections integrate inputs bilaterally and extensively from frontal and prefrontal networks.

### 3.4 Cortical input distributions reflect top‐down and bottom‐up nature of projections

We broadly categorized cortical inputs into sensory, associational, motor, prefrontal, and orbitofrontal areas to examine the distribution of functional modalities in the cortical inputs to the top‐down ACC→LP neurons and bottom‐up LP→ACC neurons. In general, LP→ACC neurons received inputs from a greater diversity of cortical areas than ACC→LP neurons. As described above, prefrontal areas dominated inputs to ACC→LP neurons. This was followed by inputs from associational areas (18.4%) (Figure 5a,c). In particular, these inputs were starkly dominated by inputs from the retrosplenial cortex, which represented almost all of the input from associational areas (Figure 5c). Inputs from primary and higher order sensory areas represented 8.4% of inputs to ACC→LP neurons (Figure 5a,e). In contrast, consistent with the bottom‐up nature of LP→ACC projections, sensory cortices represented the majority of cortical inputs, while prefrontal and associational areas contributed to 8.4% and 6.1% of total input to LP→ACC neurons (Figure 5b,d).

**FIGURE 6:**
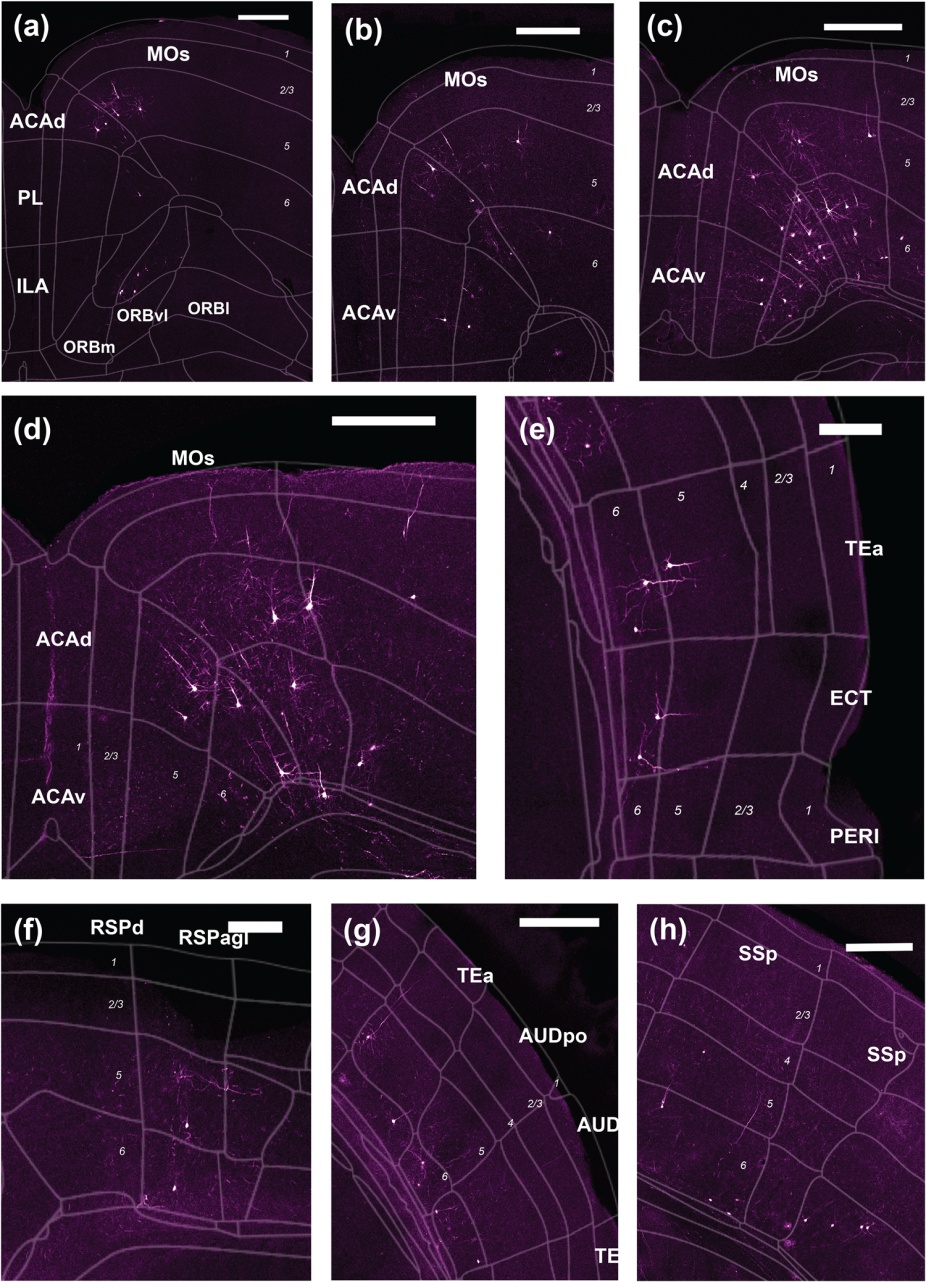
Cortical inputs to LP→ACC neurons. LP→ACC neurons receive cortical inputs from (a–d) prefrontal areas ACC, PL, and MOs, scale bars = 250 μm. (e) Temporal association cortical areas TeA and ectorhinal cortex, scale bar = 125 μm. (f) Retrosplenial areas, scale bar = 125 μm. (g) Auditory cortex and (h) somatosensory cortex, scale bars = 250 μm

Reciprocal inputs from ACC to LP→ACC neurons were found all along the anterior‐posterior axis of ACC (Figure 6a–d) and represented 5.5% of total input (Figure 5d). Most of the other prefrontal inputs originated from the secondary motor cortex (MOs) and the prelimbic cortex (PL) (Figures 5d and 6a–d), with minimal inputs from the infralimbic cortex. Associational cortex inputs to LP→ACC neurons were largely from the posterior temporal association area (TeA), ectorhinal cortex (ECT) (Figures 5d and 6e), and the retrosplenial cortices (RSP) (Figures 5d and 6f). Notably, these associational areas are all known to have visually driven activity, particularly in response to visuospatial cues and other higher order visual features (Fischer et al., 2020; Nishio et al., 2018; Powell et al., 2020). In sum, similar to ACC→LP neurons, LP→ACC neurons receive common association cortex input from retrosplenial areas, albeit to a lesser extent. Instead, LP→ACC neurons additionally received a broader range of association cortex inputs from temporal cortical areas likely involved in visuospatial and multisensory integration.

### 3.5 Asymmetric sensory cortical contributions to the reciprocal LP→ACC circuit

We next examined the sensory modalities that made up sensory cortical inputs to these projector populations. Apart from input originating from visual areas, LP→ACC neurons also received substantial cortical input from primary auditory (Figure 6g) and somatosensory areas (Figure 5h). In fact, when considering cortical areas by their dominant modality, the different sensory modalities (visual, auditory, and somatosensory) appeared to send comparable proportions of input to LP→ACC neurons (Figure 5f), arguing for a multimodal associational nature of information integrated and relayed by the medial LP region. Furthermore, inputs from the higher order sensory areas (e.g., VISpm and AUDv) were generally greater than from the primary sensory areas (VISp and AUDp). Thus, the sensory information received by LP→ACC neurons is unlikely to encode simple sensory features, and involves the integration of information from multisensory sources. In contrast, the direct sensory cortical inputs to ACC→LP neurons (Figure 5e) were dominated by visual cortices (Figure 5f), although it is likely that cross‐modal information is conveyed indirectly via associational cortex input and other ACC neurons. In summary, when considering inputs from sensory cortices, the LP→ACC pathway integrates much more multimodal input, while the ACC→LP pathway is dominated by the visual modality.

**FIGURE 7:**
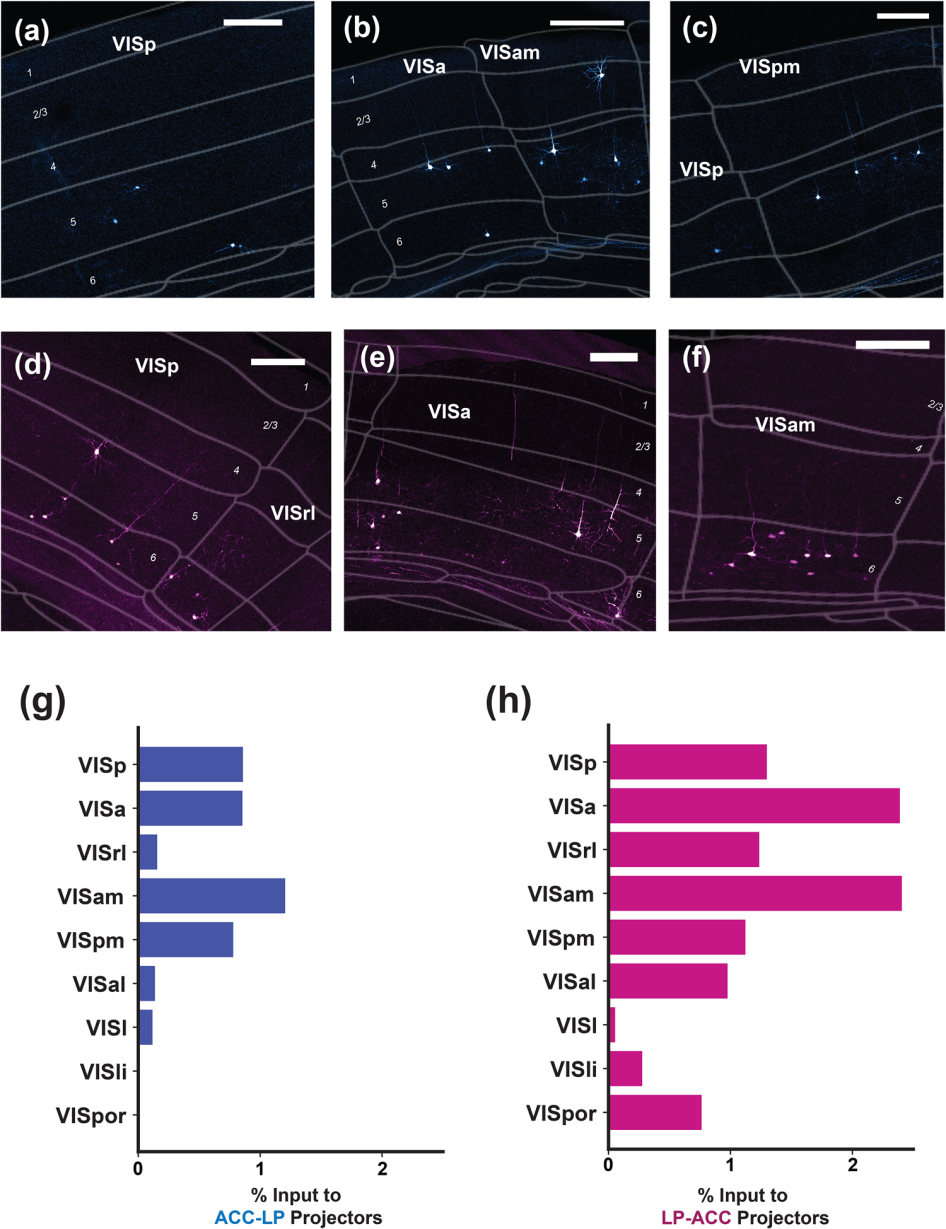
Visual cortical inputs to ACC→LP and LP→ACC projectors. (a–c) Micrographs showing inputs to ACC→LP neurons (a) VISp, (b) VISa and VISam, (c) VISp and VISpm. (d–f) Micrographs showing inputs to LP→ACC neurons (a) VISp and VISrl, (b) VISa, and (c) VISam. Scale bars = 125 μm. (g and h) Distribution of inputs from different visual cortical areas to ACC→LP (g) and LP→ACC (h) projectors

Both LP→ACC and ACC→LP neurons appeared to receive predominantly higher order visual cortical input rather than from primary visual cortex (Figure 5e,f). When comparing inputs from specific visual areas that surround VISp, ACC→LP neurons largely received input from the medial higher order visual cortical areas, predominantly VISam and VISpm (Figures 5, 7, and 7g). VISam was also the largest visual cortical input source for LP→ACC (2.3% of total input) neurons, but LP→ACC neurons also received substantial input from more higher order visual cortices than ACC→LP neurons (Figures 5d and 7d–f). Parts of VISrl and VISa overlap with a region that many rodent studies have defined as the posterior parietal cortex (Goard et al., 2016; Lyamzin & Benucci, 2019; Pho et al., 2018), and representation of these areas may reflect how the pulvinar is embedded in the frontal‐parietal network (Zhou et al., 2016). To summarize, medial higher visual areas provide greatest input to both LP‐ACC and ACC‐LP neurons, but visual cortical input to LP‐ACC neurons comes from a greater diversity of areas.

### 3.6 Subcortical inputs to ACC→LP neurons arise predominantly from areas regulating arousal and spatial cognition

**FIGURE 8:**
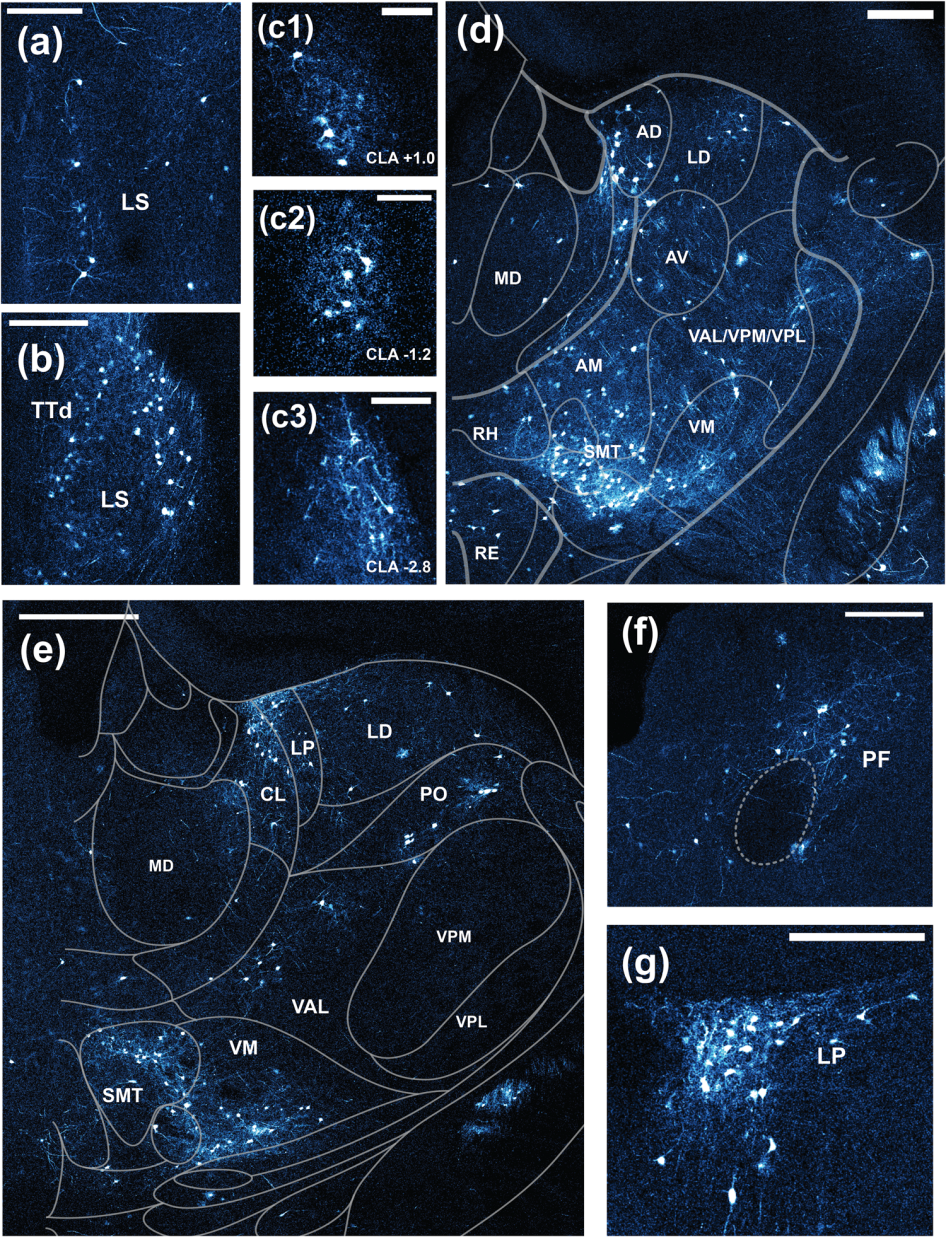
Subcortical inputs to ACC→LP neurons. (a and b) Inputs from the lateral septal area. (c1, c2, and c3) Claustral inputs span the anterior–posterior axis. Scale bars = 100 μm. (d–g) Thalamic inputs to ACC→LP neurons are largely from the anterior thalamic nuclear group, submedial thalamus (SMT), and lateral group. Scale bars (except (c)) = 250 μm

In the rostral pallidum, ACC→LP neurons receive input from the lateral septum (LS, Figure 8a,b), a limbic structure involved in the regulation of arousal, spatial cognition, as well as motivationally driven movements and actions (Wirtshafter & Wilson, 2021). From the midbrain, the dominant source of input to ACC→LP is the ventral tegmental area, indicating that the ACC→LP network also receives mesocortical (presumably dopaminergic) input.

Another input involved in arousal regulation is the claustrum (CLA), which is characterized by reciprocal connections with all cortical areas (Chia et al., 2020; Zingg et al., 2014; Zingg et al., 2018) and shares many functions also attributed to the frontal‐parietal‐pulvinar network, such as salience processing and top‐down attention (Mathur, 2014; White et al., 2018). While claustral inputs alone made up just 2.5% of total input to ACC→LP neurons, they were found consistently throughout the rostrocaudal axis in the ipsilateral hemisphere (Figure 8c1–c3).

The distribution of thalamic input to ACC→LP neurons was consistent with known thalamic inputs to the ACC itself (Zhang et al., 2016), with thalamic inputs dominated by nuclei in the anterior nuclear group (particularly the anteromedial [AM] nucleus) and the submedial thalamus (Figure 8d). Other prominent thalamic inputs included the arousal‐regulating central lateral (CL) thalamic nucleus (Redinbaugh et al., 2020; Schiff, 2008), as well as motor thalamic structures receiving basal ganglia outputs, including the lateral anterior ventral nucleus (VAL) and ventral medial thalamus (VM) (Figure 8d,e), and parafasicular thalamus (PF, Figure 8f). Reciprocal inputs from LP were present along the medial aspects in anterior LP (Figure 8g) but were not as numerous as inputs from other anterior thalamic nuclei. This highlights the fact that ACC→LP input is likely more than simple feedback of LP→ACC input. Instead, the densest thalamic input to ACC→LP neurons was from the anterior thalamic nuclei, which play important roles in regulating memory encoding and spatial cognition (Jankowski et al., 2013; Roy et al., 2021). Taken together, ACC→LP neurons integrate subcortical inputs predominantly from arousal‐regulating areas (e.g., claustrum, CL, and VM) and areas involved in memory and spatial cognition (e.g., anterior thalamic nuclei and LS), which may specify behavioral contexts for top‐down modulation.

### 3.7 Tectal input to LP→ACC neurons arises predominantly from intermediate and deep layers of SC

**FIGURE 9:**
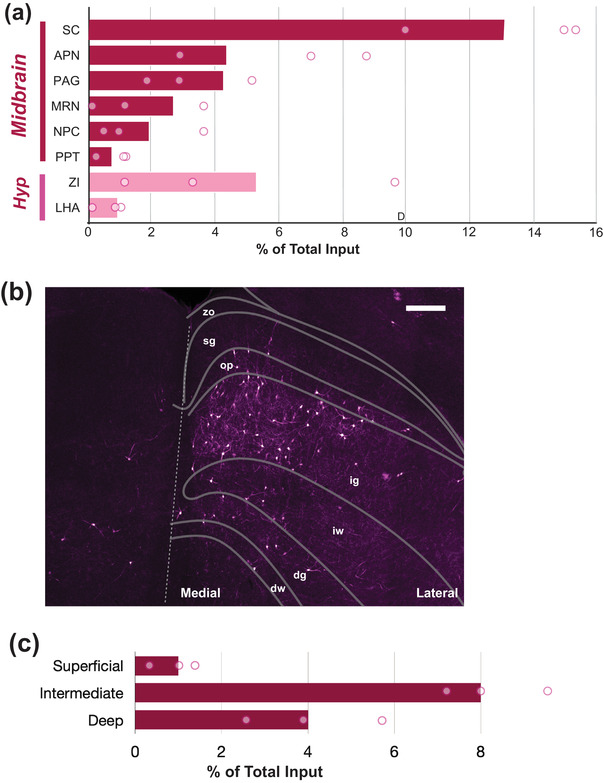
Tectal and pretectal midbrain inputs to LP→ACC neurons. (a) Distribution of midbrain and hypothalamic inputs to LP→ACC neurons. (b) Micrograph showing laminar distribution of superior colliculus inputs to LP→ACC neurons and (c) its quantification. Bars represent mean of *n* = 3 mice, and circles represent samples from individual mice. Scale bars = 250 μm

LP→ACC neurons received approximately 62% of input cells from subcortical structures; of these, the SC is the pathway's largest single subcortical input source (Figure 9a), constituting 13.2% of total anatomical input to LP→ACC neurons. The SC is made up of multiple interconnected layers composed of distinct cell types and is well‐known to be an input to the pulvinar/LP (Beltramo & Scanziani, 2019; Bennett et al., 2019; Stepniewska et al., 2009). Recent work has revealed that LP, particularly its posterior subregion, receives significant visual input from the superficial layers of the SC, which receive direct retinal input (Beltramo & Scanziani, 2019; Bennett et al., 2019). However, LP→ACC neurons are located in anterior and medial LP subregions that do not appear to receive substantial superficial SC input. As such, we explored the laminar origins of SC inputs in more detail to determine if the SC inputs were likely to be of visual origin, or from multisensory and motor‐related SC layers. With finer layer‐specific quantification of SC input, we found that more than 90% of SC inputs to LP→ACC neurons instead originated from the intermediate and deep SC layers (Figure 9b,c), which are more strongly associated with motor and multisensory functions (Lee et al., 2020). SC also has different projection zones along its mediolateral axis that differ in their complement of cortical inputs (Benavidez et al., 2021). We note that SC inputs to LP→ACC neurons were mostly found along the midline, the zone of SC that receives the most visual cortical and retrosplenial inputs (Benavidez et al., 2021). A small proportion of SC inputs were also labeled in the contralateral SC, although ipsilateral projections dominated (Figure 9b).

**FIGURE 10:**
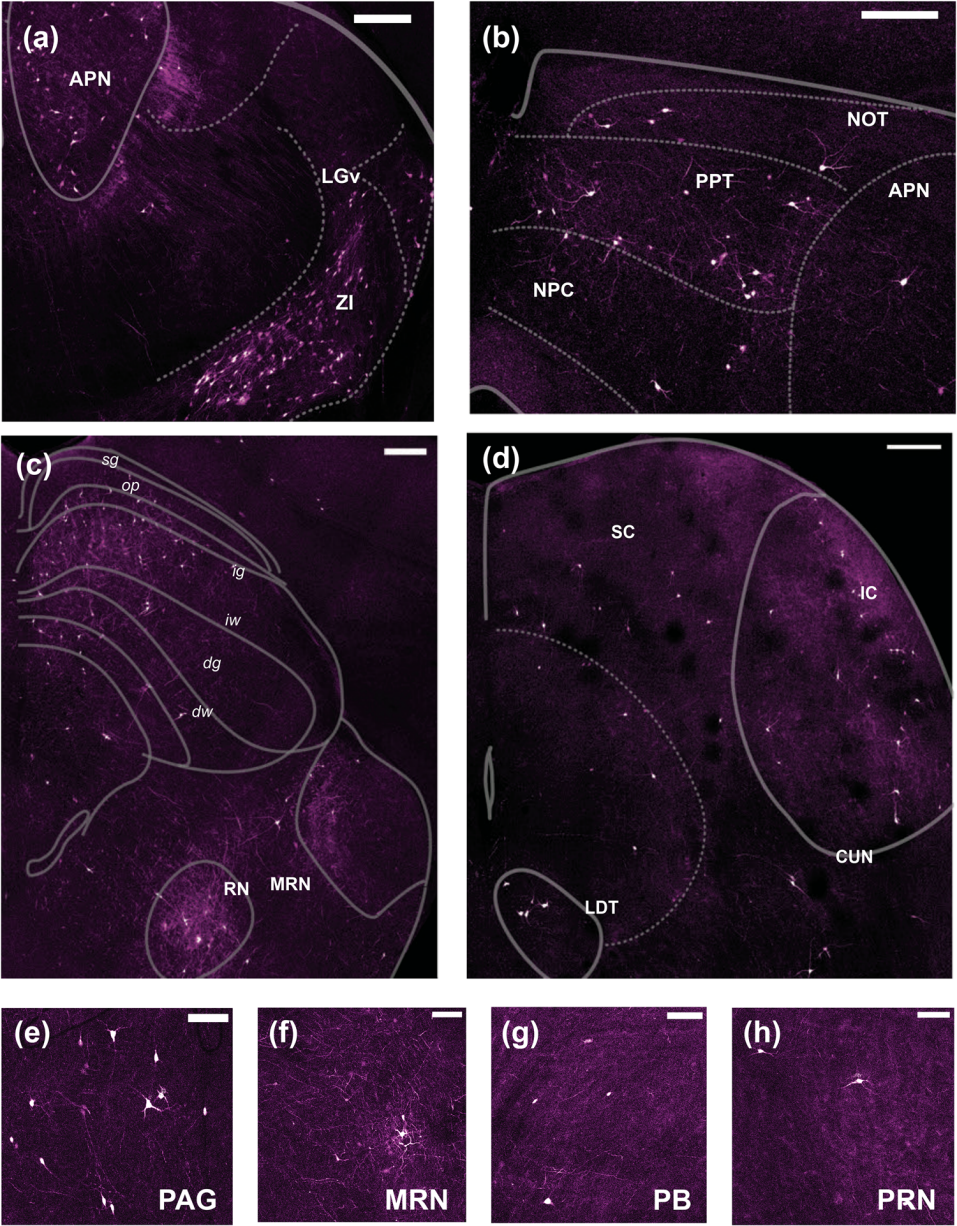
Midbrain and hindbrain inputs to LP→ACC neurons. Inputs to LP→ACC neurons from (a) ZI, LGv, and the APN, (b) pretectal areas, (c) SC and reticular formation. (d) Superior and inferior colliculi and the cholinergic laterodorsal tegmentum (LDT). Scale bars = 250 μm. (e) Periaqueductal gray (PAG), (f) midbrain reticular nucleus (MRN), (g) parabrachial nucleus (PB), and (h) pontine reticular nucleus (PRN). Scale bars = 100 μm

Within the thalamus, LP→ACC neurons received inputs from TRN and the ventral lateral geniculate nucleus (LGv) (Figure 10a), which likely serves as intrathalamic sources of inhibition (see next section). LP→ACC neurons also received abundant inputs from the pretectal areas, specifically the anterior pretectal nucleus (APN), nucleus of the posterior commissure (NPC), and posterior pretectal nucleus (PPT) (Figure 10a,b). These pretectal areas receive direct retinal input and are involved in the control of oculomotor functions and ocular reflexes, such as the pupillary light reflex and optokinetic reflex (Carpenter & Pierson, 1973; Levine & Schwartz, 2020; Masseck & Hoffman, 2009). In the caudal midbrain and hindbrain, LP→ACC neurons received some sparse input from the periaqueductal gray (Figure 10c–e). Sources of cholinergic inputs, such as the laterodorsal tegmental nucleus (LDT, Figure 10d) to the thalamus, also send input to LP→ACC (Huerta‐Ocampo et al., 2020). LP→ACC neurons also receive input from the midbrain reticular nucleus (Figure 10f) that controls motor functions, including, but not limited to, eye movements. Pontine areas send sparse input to LP→ACC neurons from the parabrachial nucleus (PB, Figure 10h) and the pontine reticular formation (PRF, Figure 10h). Thus, the mid and hindbrain inputs to LP‐ACC neurons appear to predominantly be sources of oculomotor and other movement‐related activity.

### 3.8 Subcortical inputs to LP→ACC neurons include long‐range inhibitory sources

As a higher order thalamic nucleus, LP is expected to receive its driving input from the cortex (Bickford, 2016; Guillery & Sherman, 2002). Consistent with this notion, LP firing is strongly suppressed during cortical silencing of the visual cortices (Beltramo & Scanziani, 2019; Bennett et al., 2019). However, we found that the number of subcortical inputs outnumbered cortical inputs to LP→ACC neurons by about 20% (Figure 2e). This is likely explained by the contribution of numerous modulatory subcortical inputs with weak excitatory drive. Alternatively, it is possible that some of these subcortical inputs instead provide inhibitory input.

Indeed, a major source—the second largest subcortical source—of input to LP→ACC neurons was the zona incerta (ZI) (8A, 9A), a subthalamic structure that sends strong GABAergic inputs to higher order thalamic nuclei (Bartho et al., 2002). As such, we asked whether other subcortical regions provided inhibitory inputs to LP‐ACC neurons.

**FIGURE 11:**
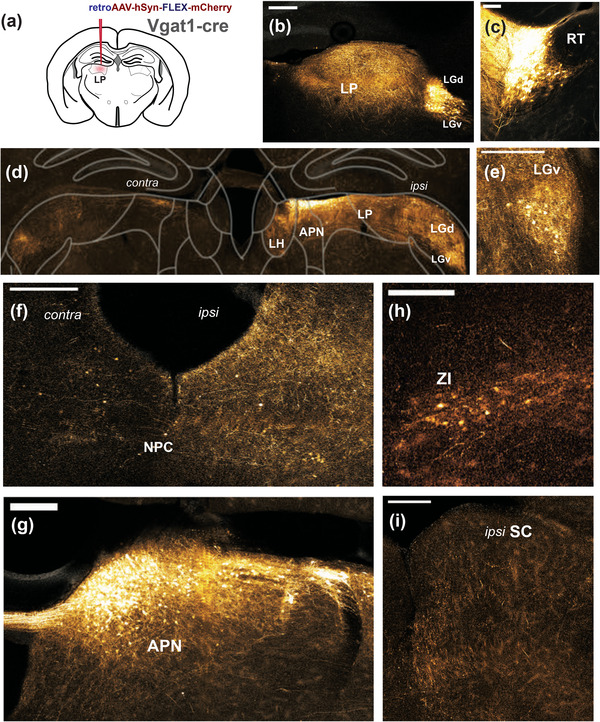
Inhibitory (Vgat1‐positive) inputs to LP. (a) Retrograde labeling of inhibitory inputs to LP, with injection of AAV carrying Cre‐dependent fluorescent tracer in LP of a Vgat1‐Cre mouse. (b) Vgat1‐positive axons in LP, and cell bodies in LGv. (c) Intrathalamic inhibition from the thalamic reticular nucleus (RT). (d) Vgat1‐positive cell bodies in ipsilateral APN and LGv. Sparse interneuronal labeling in LP and LGd. Vgat1‐positive axons in contralateral thalamus. (e) Vgat1‐positive inputs from LGv. (f) Sparse inhibitory inputs from both hemispheres of the nucleus of the posterior commisure (NPC). (g) Vgat1‐positive inputs from the anterior pretectal nucleus (APN). (h) Inhibitory inputs from the zona incerta. (i) Vgat1‐positive axons but not cell bodies in ipsilateral SC. Scale bars = 250 μm

As our projection‐specific rabies tracing strategy did not allow us to distinguish between excitatory and inhibitory input cell types, we performed a separate set of anatomical tracing of inputs to LP, specifically in Vgat1‐Cre mice. To target Vgat1‐positive inhibitory inputs to LP, we injected retrograde AAV carrying Cre‐dependent fluorophore, mCherry (Figure 11a). Several subcortical structures identified as inputs to LP→ACC neurons were also labeled as Vgat1‐positive inputs to LP. As expected, within the thalamus, the inhibitory thalamic reticular nucleus (RT) was labeled as an inhibitory source (Figure 11c). Inhibitory inputs were also found to originate from the ventral LGN (LGv, Figure 11b,d,e). In addition, sparse Vgat1‐positive cell bodies were observed in LP and dorsal LGN (LGd), particularly along the dorsal aspects within the nuclei. These are likely thalamic inhibitory interneurons, which are relatively sparse and typically restricted to visual nuclei in the rodent thalamus (Evangelio et al., 2018). Thus, intrathalamic inhibition of LP neurons can come from RT, LGv, and to a more limited extent, from local LP interneurons. Vgat1‐positive cell bodies in the thalamus were found almost exclusively in the hemisphere ipsilateral to the retrograde injection. However, at least some of the brain‐wide inhibitory inputs to LP also project to the contralateral thalamus, as Vgat1‐positive axon collaterals could be seen in the contralateral hemisphere (Figure 11d).

Other areas identified as potential sources of inhibitory inputs to LP→ACC neurons included pretectal areas, such as the NPC (Figure 11f) and APN (Figure 11g) in addition to the zona incerta (ZI, Figure 11h), where GABAergic neurons are the major cell type (Giolli et al., 1985). While the SC is also a potential source of long‐range inhibitory input (Takahashi et al., 2005), we found that the SC contained only Vgat1‐positive axon collaterals but not cell bodies (Figure 11i), suggesting that SC input to LP is likely not inhibitory. The presence of axonal labeling in SC also indicates that some of the inhibitory projections to LP collateralize beyond the thalamus to include the ipsilateral SC. Together, these findings reveal several specific long‐range inhibitory inputs to LP that potentially shape its functional responses, including those conveyed to ACC.

## 4 DISCUSSION

**FIGURE 12:**
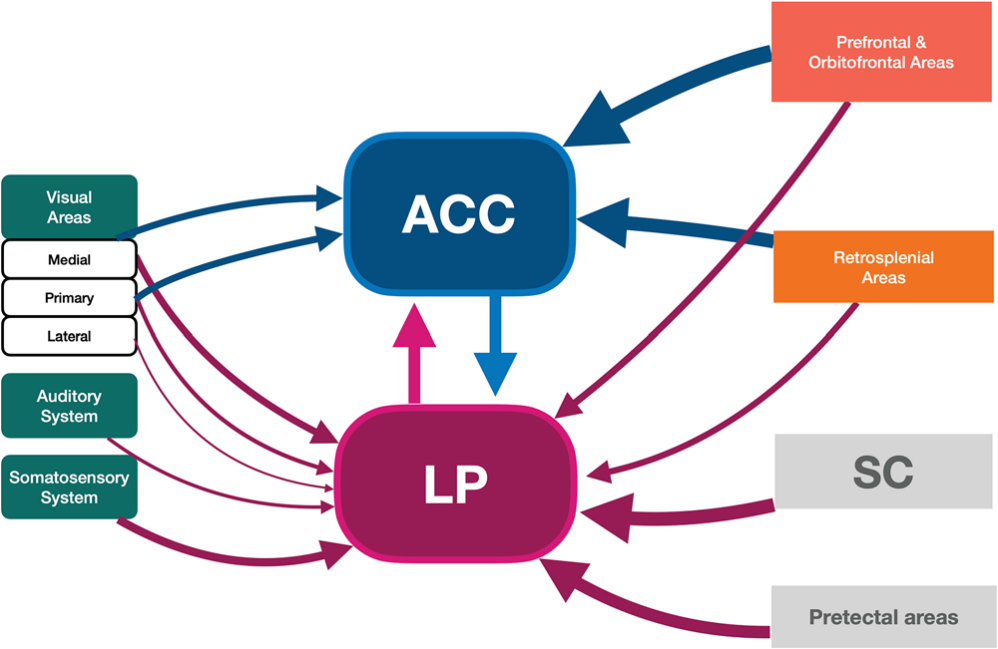
Overview of inputs to ACC→LP and LP→ACC neurons. Blue arrows represent inputs to ACC→LP neurons. Magenta arrows represent inputs to LP→ACC neurons. Weight of the arrows reflects projection‐specific relative proportion of inputs (not to scale) and should only be compared within projections (blue for ACC →LP and magenta for LP→ACC)

Our study provides a comprehensive anatomical overview of inputs integrated by the rodent reciprocal LP→ACC circuit (Figure 12). The pulvinar/LP has been established as a hub‐like structure with extensive reciprocal connectivity with many cortical regions (Bennett et al., 2019; Bridge et al., 2016; Kaas & Lyon, 2007; Shipp, 2003). Our brain‐wide mapping of inputs to the circuit establishes not only the cortical areas that can influence LP→ACC interactions but also highlights the important subcortical contributions. These data position LP as a site of convergence for both cortical and subcortical inputs that is conveyed not just to the sensory cortices but also to prefrontal areas such as the ACC to potentially exert top‐down influence (Bollimunta et al., 2018; Hu et al., 2019). The inputs integrated by the ACC→LP pathway, particularly the proportion of prefrontal and frontal inputs, suggest strong incorporation of prefrontal local computations with a role for top‐down priorities conveyed by the ACC. These behavioral priorities also likely incorporate spatial contexts (Aggleton et al., 2010) from another strong source of input to ACC→LP neurons, the retrosplenial areas. ACC→LP projections also receive abundant interhemispheric inputs locally within ACC and from other prefrontal areas. In turn, the LP relays predominantly sensorimotor and multimodal information to ACC. This is evident from the extensive inputs from sensorimotor‐associated layers and zones of the SC, pretectal areas, as well as inputs from sensorimotor cortices, such as the parietal areas (including VISa and VISam) and the retrosplenial cortex. Higher order sensory information from these areas may combine with inputs about eye and orienting movements, from the tectal and pretectal areas, to contribute to specifying spatial contexts for modulation and coordinating spatial selective attention.

### 4.1 Recurrent local ACC inputs to ACC→LP neurons

ACC→LP neurons were found to have many local inputs from within ACC, with many found in the contralateral ACC, suggesting that top‐down ACC→LP input incorporates information from both hemispheres. We also observed that the contralateral ACC inputs mostly clustered in the ventral ACC. Although we lack consensus regarding rodent ACC and its subdivisions (van Heukelum et al., 2020), cortical connectivity‐based models suggest the ventral ACC subdivision, as mapped to the Allen Reference Atlas, may be additionally involved in a medial subnetwork important for relaying spatial information from the dorsal subiculum to the mPFC (Zingg et al., 2014). Models of pulvinar function show that interhemispheric competition can explain some deficits in distractor filtering following pulvinar lesions (Jaramillo et al., 2019); unilateral pulvinar microstimulation leads to cortical activation in both hemispheres (Kagan et al., 2021), suggesting that pulvinar activation is likely to involve both hemispheres and its function involves cross‐hemispheric communication. Interhemispheric interaction of ACC and ACC→LP projections is particularly interesting in context of work showing that transcranial magnetic stimulation of the frontoparietal area biases spatial attention to shift to the contralateral visual space (Kinsbourne, 1977; Szczepanski & Kastner, 2013). Furthermore, hemispatial neglect of contralesional space due to unilateral pulvinar inactivation could be partly alleviated by the presence of an asymmetrically larger reward in the contralesional space (Wilke et al., 2013), highlighting the powerful role of top‐down modulation in prioritizing visual space. Further investigation into ACC as a source of interhemispheric information incorporated into pulvinar circuits is likely to be an important step forward in understanding the control of spatial attention.

### 4.2 Asymmetric representation of sensory modalities in LP→ACC circuits

The top‐down ACC→LP projection and bottom‐up LP→ACC projection differ in the sensory modalities represented in their input. When considering only inputs along the sensory domain, LP→ACC neurons receive input from broadly multisensory cortical and subcortical areas, whereas ACC→LP neurons receive predominantly visual input. We find that the higher visual cortical input to LP→ACC neurons is consistent with previous reports of medial LP neurons generally having broader visual tuning to oriented gratings but greater responsiveness to complex visual stimuli, such as pattern motion (Foik et al., 2020). Compared to the range of visual cortical inputs to LP→ACC neurons, ACC→LP neurons receive visual cortical activity from more restricted areas, specifically VISp, VISam, and VISpm. As such, the LP input to ACC is able to provide ACC with information from a greater range of visual cortical areas. Activation of ACC→LP and ACC→SC projections improves performance in mice performing a visual discrimination task (Hu et al., 2019), whereas suppressing ACC‐SC activity improves contraversive orienting behavior in mice performing ball rotations in response to visual stimuli in the contralateral or ipsilateral hemifield (Huda et al., 2020). Downstream ACC projections thus link vision to action, and the role of ACC‐LP projections may be to provide top‐down signals for action selection.

The LP→ACC projection is more multimodal and did not exhibit a strong bias for visual inputs over other sensory modalities. In nonhuman primates, the medial pulvinar is the most broadly multisensory pulvinar subregion, with examples of responsiveness to complex visual, auditory, and somatosensory stimuli (Gattass et al., 1978; Homman‐Ludiye & Bourne, 2021; Yirmiya & Hocherman, 1987). The somatosensory system appears to be strongly represented among LP→ACC inputs and could reflect the embedded functions of the parietal cortices in this network, where there is also integrated processing of multimodal information (Mohan et al., 2019; Runyan et al., 2017). In human subjects, impaired performance in a visual target detection task under sedation with noradrenergic a2 agonists is partly rescued by the presence of loud auditory white noise in a manner that appears dependent on medial pulvinar activation (Coull et al., 2004). The cross‐modal interaction may not be restricted to visual perception, as rodent LP activation has also been found to sharpen auditory frequency tuning in primary auditory cortex, particularly in the presence of noise (Chou et al., 2020).

### 4.3 LP as a bottom‐up integrator of subcortical inputs

Although the distribution of cortical inputs to LP→ACC neurons shows that higher sensory and associational cortical information is integrated by these neurons, LP→ACC neurons still receive substantial subcortical input, some of which provide inhibitory control.

The largest extrathalamic inhibitory source to LP→ACC neurons is the ZI, which has direct reciprocal connections with the cortex (Chen & Kriegstein, 2015) and the SC (May & Basso, 2018). The ZI is a heterogeneous structure, and the GABAergic projections from ZI have been associated with various approaches and avoidance behaviors (Ahmadlou et al., 2021; Chou et al., 2018; Zhao et al., 2019). ZI projections to a closely related thalamic nucleus, the POm, have been shown to gate somatosensory activity in POm (Trageser & Keller, 2004; Trageser et al., 2006) and regulate nocifensive behaviors (Wang et al., 2020). ZI projections to pulvinar/LP have been much less studied, but will be an interesting avenue for future research. The ZI also has extensive connections with another major source of inhibitory inputs to LP→ACC neurons, the APN (Giber et al., 2008), and receives cholinergic inputs from brainstem PPN and LDT that can cause thalamic disinhibition via the ZI (Trageser et al., 2006). Thus, the subcortical inputs to LP→ACC neurons are extensively interconnected with multiple paths to LP. We note that these subcortical inputs to LP→ACC neurons are also not unique to this projection, and have also been identified in LP inputs to VISal and VISpm (Blot et al., 2021), and are thus likely to influence LP activity as a whole.

### 4.4 Collicular influences on LP–ACC interactions

We found that the SC is the single largest subcortical source of anatomical input to LP→ACC neurons. However, unlike other LP subregions (Beltramo et al., 2019; Bennett et al., 2019), these collicular inputs originate not from the superficial retino‐recipient layers of SC but from medial intermediate and deep layers. In primates, intermediate and deep SC also serve as the primary source of subcortical input to the medial pulvinar (Benevento & Standage, 1983). Notably, SC inputs to LP→ACC neurons are mostly found along medial SC. Interestingly, this SC projection zone receives a distribution of cortical inputs that corresponds to visuomotor areas with the highest density of inputs from visual cortical areas, ACC, RSC, TeA, and parietal inputs (Benavidez et al., 2021)—all major areas embedded in the bidirectional LP‐ACC network we delineated.

Intermediate and deep layers of SC are not directly retino‐recipient, and also have responses to a greater range of sensory modalities (Ito et al., 2017; Lee et al., 2020). The SC plays a major role in orienting and reaching movements, with eye and head orienting movements being of particular relevance to the pulvinar circuitry (Gandhi & Katnani, 2011; Zénon & Krauzlis, 2012; Basso & May, 2017; Huda et al., 2020). ACC inputs to superficial SC modulate visual responses in V1 (Hu et al., 2019), while ACC inputs to intermediate and deep SC projections decrease contraversive and increase ipsiversive actions (Huda et al., 2020). Since the SC does not project directly to ACC, the LP may serve as one of the feedback pathways for ACC‐SC circuits. Multiple human and nonhuman primate studies implicate the pulvinar, SC, FEFs, and ACC together in attentional orienting (Rafal & Posner, 1987) and oculomotor functions (Schneider et al., 2020). Together with the abundance of pretectal inputs to LP→ACC neurons, it is likely that intermediate and deep layer SC inputs to medial LP in rodents are also involved in visuomotor orienting. Beyond orienting, the intermediate layer SC input to LP has been identified to be critical for mediating behavioral responses to visually evoked threat (Wei et al., 2015), with LP serving a critical relay for medial (but not lateral) intermediate SC layer input to the lateral amygdala. Deep and intermediate SC input on innate visual threats may also be relayed to ACC via the LP to serve as a rapid parallel pathway for the salient visual threat before various steps of visual processing, to signal different attentional priorities or coordinate orienting eye movements toward the threat.

### 4.5 Technical considerations

Our study mapped inputs to ACC→LP and LP→ACC projector populations by aligning DAPI‐stained imaged coronal brain sections to the Allen Reference Atlas using cytoarchitectonic landmarks. Cell counts within regional boundaries and cortical laminar assignments were defined by alignments to the atlas which represent an average across many mice. Boundaries, such as those between individual thalamic nuclei and between different visual cortical areas, nonetheless vary across individual mice. Precise assignment of regional boundaries can only be achieved by further functional or molecular characterization. As such, we caution that cell counts between tightly adjacent regions may be inevitably conflated and more challenging to compare.

Although ACC→LP projections originate from both layers 5 and 6, it is important to point out that tropism of retrograde‐AAVs used in our rabies tracing approach preferentially transfects layer 5 over layer 6 neurons (Figure 1c,d). Thus, the mapped inputs to ACC→LP starter cells likely represent inputs to a greater proportion of layer 5 than to layer 6 ACC→LP neurons. As corticothalamic projections from layers 5 and 6 can have different functional impact on thalamic neurons (Kirchgessner et al., 2020; Kirchgessner et al., 2021; Usrey & Sherman, 2018), it will also be important for future work to compare how inputs to top‐down ACC→LP drivers and modulators may differ. Indeed, layer 5 ACC→LP neurons may also have collaterals in other subcortical structures, such as the SC (Hu et al., 2019), while layer 6 projections are exclusively corticothalamic—these laminar‐defined projections could be embedded in distinct interacting subnetworks.

Although our study mapped inputs specific to ACC→LP and LP→ACC projector populations, each of these projections may also collateralize to multiple downstream targets. For example, at least some of layer 5 ACC→LP neurons also simultaneously project to SC (Hu et al., 2019), while LP neurons projecting to ACC often also extend axons to multiple other cortical regions, particularly medial higher visual areas (Nakamura et al., 2015). As such, the inputs integrated will also likely impact a larger network to which the neurons simultaneously project. The multiple parallel pathways embedded in the ACC→LP network could be critical in coordinating synchronous activity across areas and speak to the integrated nature of their proposed functions.

Finally, we note that in our ACC→LP experiment, we found sparse artifactual labeling of some midbrain sources such as the SC that are not known to have direct cortical projections. It is possible that anterograde transduction of AAV helper viruses from ACC to thalamic regions could lead to small numbers of starter cells outside of the cortex. Our control experiments where transsynaptic spread is prevented by providing no G protein complementation (Figure 3a) indicated no labeled cells outside of the starter cell region, suggesting that glycoprotein‐independent labeling occurs at an extremely low rate, if at all. Furthermore, the rabies‐infected cells found in the midbrain were considerably sparser in contrast to midbrain inputs to LP→ACC. Thus, we expect that our results predominantly reflect inputs to ACC→LP starter neurons.

## 5 CONCLUSIONS

We present in this study a comprehensive projection‐specific monosynaptic mapping of inputs to the reciprocal LP and ACC network in mice. We show that the LP‐ACC network integrates information from regions which parallel that of the primate pulvinar‐prefrontal network. Much like the primate medial pulvinar, mouse LP→ACC neurons in the medial LP also incorporate multimodal inputs from many different sensory domains, although sensory input to ACC→LP neurons appears to be predominantly visual. This circuit in mice may serve as an evolutionary roadmap for pulvinar–prefrontal interactions that control advanced visuomotor capacities in primates. We also identify several common sources of inputs to LP→ACC and ACC→LP neurons from regions involved in orienting and sensorimotor control. Finally, our findings highlight that interhemispheric interactions of top‐down ACC→LP inputs may be an important contributor to visuospatial attention. In sum, our study serves as a primer for future circuit‐level examination of LP–ACC interactions and inputs integrated by this circuit.

## FUNDING

Supported by an A*STAR (Singapore) National Science Scholarship (YNL), and grants from the NIH (R01MH126351 and R01EY028219) and the Picower Institute Innovation Fund (MS).

## CONFLICT OF INTEREST

The authors declare no conflict of interest.

## AUTHOR CONTRIBUTIONS

YNL and BZ performed the experiments and analyzed the data. YNL prepared figures and drafted the manuscript. HS prepared key viral reagents for the experiments. AB provided technical support for histology. IW provided key viral reagents and discussion during planning of the study. MS edited the manuscript and supervised the entire study.

### PEER REVIEW

The peer review history for this article is available at https://publons.com/publon/10.1002/cne.25317.

## Data Availability

The data that support the findings of this study are available from the corresponding author upon reasonable request.
